# Clinical feasibility of gastric endoscopic submucosal dissection in patients on glucocorticoids or immunomodulators: Propensity-score-matched study

**DOI:** 10.1055/a-2733-1229

**Published:** 2025-11-11

**Authors:** Hiroki Fukuya, Eikichi Ihara, Yoichiro Iboshi, Yorinobu Sumida, Daisuke Yoshimura, Shohei Hamada, Taisuke Sasaki, Akito Ohkubo, Shuichi Itonaga, Hitoshi Homma, Ryota Okitsu, Akihisa Ohno, Mitsuru Esaki, Naohiko Harada

**Affiliations:** 1 37085 Department of Gastroenterology, National Hospital Organisation Kyushu Medical Center, Fukuoka, Japan; 2 Institute of Research Center, National Hospital Organization Kyushu Medical Center, Fukuoka, Japan; 3 12923 Department of Medicine and Bioregulatory Science, Graduate School of Medical Sciences, Kyushu University, Fukuoka, Japan; 4 Department of Gastroenterology, Kitakyushu Municipal Medical Center, Fukuoka, Japan

**Keywords:** Endoscopic ultrasonography, Gastric cancer, Endoscopy Upper GI Tract, Endoscopic resection (ESD, EMRc, ...), Quality and logistical aspects, Performance and complications

## Abstract

**Background and study aims:**

Evidence on gastric endoscopic submucosal dissection (ESD) under glucocorticoids or immunomodulators (GC/IM) is limited. We evaluated whether GC/IM use affects gastric ESD outcomes.

**Patients and methods:**

We retrospectively analyzed 411 consecutive ESDs (April 2017-April 2022). GC/IM users (n = 32) were compared with controls (n = 379); 1:3 propensity-score matching yielded 27 vs 81 patients. The primary outcome was overall complications, defined as a composite of pain, fever, delayed bleeding, and delayed perforation. Secondary outcomes were each component, intra-procedure perforation, hospital stay, and use of symptom-directed treatments (analgesics, antipyretics, antibiotics).

**Results:**

Overall complications were more frequent with GC/IM than controls (44.4% vs 21.0%; *P* = 0.024; relative risk [RR] 2.11, 95% confidence interval 1.16–3.84), driven by pain (40.7% vs 18.5%; *P* = 0.035) and fever (11.1% vs 1.2%; *P* = 0.047). Rates of major complications did not differ (delayed bleeding 3.7% vs 2.5%; delayed perforation 0% vs 0%). Intra-procedure perforation was numerically higher (7.4% vs 3.7%) without significance. Median (interquartile range) hospital stay showed a small, non-significant difference (9 [7–12] vs 8 [7–9] days; *P* = 0.064). Symptom management was used more often with GC/IM (analgesics 25.9% vs 3.7%, *P* = 0.002; antipyretics 7.4% vs 2.5%, *P* = 0.270; antibiotics 7.4% vs 3.7%, *P* = 0.597).

**Conclusions:**

In patients receiving GC/IM, gastric ESD was associated with a higher incidence of minor, clinically managed events—chiefly pain and transient fever—whereas major complications remained uncommon. With close monitoring and prompt symptom-directed care, gastric ESD appeared clinically feasible, albeit with slightly greater resource use and observation time.

## Introduction

Endoscopic submucosal dissection (ESD) has become widely accepted as an effective treatment for early-stage gastric cancer, providing high en bloc resection rates with relatively low complication rates 1 . Compared with gastrectomy, ESD is less invasive and places a lower physical burden on patients 2 3 . Consequently, ESD has become a viable alternative to surgery for high-risk patients, such as older individuals 4 or those with significant comorbidities 5 , potentially offering fewer complications than gastrectomy.

Despite these positive findings, patients with comorbid conditions exhibit varying disease severity and different types of systemic involvement, which may differentially influence risk of post-ESD complications. One study reported that ESD is effective across different American Society of Anesthesiologists Physical Status (ASA-PS) categories, but that higher ASA-PS scores correlate with more frequent complications 6 . However, other studies have found no significant increase in adverse events (AEs) despite varied comorbidity profiles 4 5 . These discrepancies suggest that particular subgroups of patients may warrant closer attention.

One such subgroup is immunocompromised individuals, especially those receiving glucocorticoids (GCs) or immunomodulators (IMs). Commonly prescribed for chronic inflammatory diseases, GCs have been associated with an increased risk of gastric ulcers 7 , and this risk becomes even more pronounced when combined with non-steroidal anti-inflammatory drugs (NSAIDs) 8 9 . GC use is also associated with a higher risk of gastrointestinal bleeding and perforation in hospitalized patients 10 . Indeed, in colonic ESD, patients taking GCs or IMs have demonstrated an increased risk of post-coagulation syndrome 11 . These findings raise the concern that a similar pattern of complications, such as post-procedure pain, fever, delayed bleeding, and delayed perforation, could also occur following gastric ESD. In light of these considerations, it is crucial to clarify the influence of GCs and IMs on complication rates associated with gastric ESD.

This information is essential for optimizing procedural techniques and for postoperative management in this growing patient population. We hypothesized that patients receiving GC/IM therapy would experience a modest increase in overall incidence of clinically managed post-ESD events, whereas major complications (delayed bleeding or perforation) remained uncommon and could be managed with standard rescue measures. Therefore, this study aimed to elucidate whether treatment with GCs or IMs increases risk of post-gastric ESD complications, thereby providing valuable insights to improve the care of immunocompromised patients undergoing gastric ESD.

## Patients and methods

### Study design and patients

**Fig. 1:**
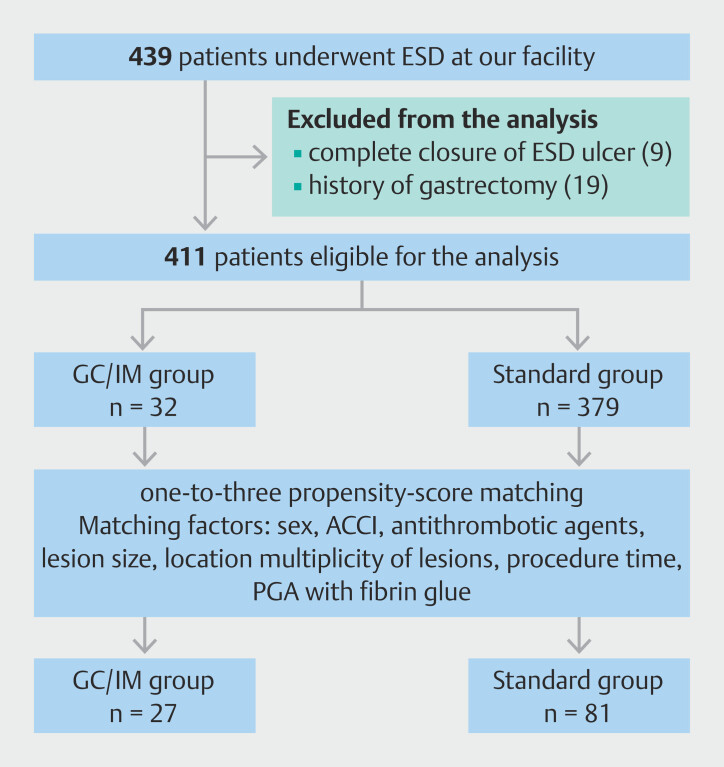
Flowchart of patient selection and propensity-score matching. Of 439 patients undergoing gastric ESD, 28 were excluded (remnant stomach, n = 19; complete closure of the post-ESD ulcer, n = 9), leaving 411 eligible patients. Of these, 32 received GC/IM and 379 did not. One-to-three propensity-score matching (covariates: sex, ACCI, antithrombotic use, lesion size, location, multiplicity, procedure time, and use of PGA sheets with fibrin glue) yielded matched cohorts of 27 GC/IM and 81 standard patients. ACCI, age-adjusted Charlson Comorbidity Index; PGA, polyglycolic acid.

This retrospective, single-center, observational cohort study was conducted using data from patient medical records and the endoscopic database at our institution. Between April 2017 and April 2022, we identified 439 consecutive patients who underwent gastric ESD. Of these, 19 patients who underwent ESD for remnant stomach and nine patients who underwent complete closure of the post-ESD ulcer were excluded because these factors could confound the evaluated effects of GCs or IMs on ESD complications. Consequently, 411 patients were included in the final analysis. Patients receiving either GCs or IMs were classified as the GC/IM group (n = 32), and patients without such treatments were allocated to the standard group (n = 379). The patient selection process and group allocation are summarized in Fig. 1 . This study was reviewed and approved by the institutional review board of our hospital (approval number: 23C008). The study adhered to the ethical principles of the Declaration of Helsinki.

### ESD procedure

All ESD procedures were performed by 17 therapeutic endoscopists: six experts (each with ≥ 100 gastric ESD cases) and 11 non-experts (< 100 cases each) under expert supervision, and some cases were co-performed by an expert and a non-expert operator. Procedures were performed on hospitalized patients under sedation with midazolam and dexmedetomidine, and analgesia with meperidine using a standard single-channel endoscope (GIF-Q260J or GIF-290T; Olympus, Tokyo, Japan). An electrosurgical generator (VIO300D or VIO3; ERBE, Tübingen, Germany) was used in combination with a Dual Knife (2.0 mm; Olympus, Tokyo, Japan) or a Clutch Cutter Long (Fujifilm, Tokyo, Japan). A bipolar coagulation device (Hemo-Stat Y; Pentax) was used for hemostasis during the procedure. For submucosal injection, physiological saline, 20% concentrated glycerin-fructose (Glycerol; Chugai Pharmaceuticals, Tokyo, Japan), or 0.4% sodium hyaluronic acid (Mucoup; Johnson & Johnson, Tokyo, Japan) was administered at the discretion of the endoscopist. Antithrombotic management followed established institutional guidelines: low-dose aspirin was continued peri-procedurally, whereas other antiplatelet agents were switched to low-dose aspirin. Patients on warfarin underwent heparin bridging therapy until 2017; thereafter, warfarin was continued within a therapeutic prothrombin time/international normalized ratio range of 2.0 to 3.0. Direct oral anticoagulants were discontinued on the day of the procedure and resumed 1 to 2 days post-procedure if no bleeding occurred. All patients received vonoprazan (20 mg/day), a potassium-competitive acid blocker (P-CAB), both before and after ESD for a total of 8 weeks. In the event of intraoperative perforation, initial management consisted of endoscopic clip closure, followed by application of polyglycolic acid (PGA) sheets with fibrin glue if necessary. PGA sheets were also applied prophylactically when the muscularis propria was significantly exposed or injured, even in the absence of full-thickness perforation, to support tissue healing. If endoscopic intervention was insufficient, urgent surgical consultation was arranged. Second-look endoscopy was not routinely performed but was conducted selectively in cases of clinically high risk of delayed bleeding based on the operator`s decision.

### Data collection

Demographic and clinical data from the patients were collected, including age, sex, body mass index (BMI), ASA-PS, and age-adjusted Charlson Comorbidity Index (ACCI) 12 , as well as use or non-use of GCs and IMs. IMs were defined as thiopurines (e.g., azathioprine), methotrexate, calcineurin inhibitors (e.g., tacrolimus), and cytokine-targeted biologics (e.g., anti-tumor necrosis factor agents), alone or in combination. Use of antithrombotic drugs was also documented. Lesion characteristics, including size, location, morphology, and multiplicity, were recorded. In addition, procedure details, such as procedure time, presence of submucosal fibrosis, injury to the muscularis propria, and application of PGA sheets with fibrin glue, were collected. We also recorded post-procedure symptom management, including administration of analgesics, antipyretics, and antibiotics, and whether second-look endoscopy was performed.

### Measured outcomes

The primary outcome was the overall rate of post-ESD complications, defined as the combined incidence of post-ESD pain, fever, delayed bleeding, and delayed perforation. We selected a composite outcome to capture the full post-procedure workload, because even minor events (pain, fever) routinely prompt additional monitoring, imaging, or analgesic use. Secondary outcomes included individual rates of these post-ESD complications, incidence of intraoperative perforation, and length of hospital stay. Post-ESD pain was defined as any abdominal pain reported by patients at any time after ESD until discharge. Post-ESD fever was defined as a body temperature ≥ 37.5°C persisting from time of the ESD procedure until discharge. Delayed bleeding was defined as overt bleeding requiring emergency endoscopic hemostasis or transfusion, or a decrease of ≥ 2 g/dL in hemoglobin concentration following ESD. Delayed perforation was defined as perforation occurring ≥ 6 hours after ESD, without evidence of intraoperative perforation during the procedure. In cases in which multiple lesions were treated, characteristics and treatment specifics were recorded on the basis of the predominant lesion.

### Statistical analysis

Propensity scores for GC/IM exposure were estimated using multivariable logistic regression including sex, ACCI, antithrombotic use, lesion size and location, lesion multiplicity, procedure time, and use of PGA sheets with fibrin glue. Patients were matched 1:3 (GC/IM: standard) by nearest-neighbor matching within a caliper of 0.2 standard deviations of the logit propensity score 13 . Covariate balance after matching was assessed using the absolute standardized difference (ASD), with ASD < 0.10 considered acceptable 14 . We deliberately excluded age, daily NSAID use, and operator expertise because age is captured by the ACCI, NSAIDs were uniformly withheld peri-procedurally per institutional protocol, and all procedures (including trainee cases) were performed under direct expert supervision. When modelling exposure status (GC/IM vs standard), receiver-operating-characteristic (ROC) analyses for individual continuous covariates indicated limited predictive ability (area under the ROC curve [AUROC] ACCI 0.73; lesion size 0.48; procedure time 0.57); therefore, we applied clinically validated thresholds to dichotomize these variables: ACCI ≥ 4 (moderate-to-severe comorbidity) 15 , lesion diameter > 20 mm (odds ratio [OR] 2.70 for post-procedural bleeding) 16 , and procedure time ≥ 60 minutes (OR 2.05 for higher bleeding rates) 16 . The overall propensity-score model showed good discrimination (AUROC: 0.79, 95% confidence interval [CI] 0.70–0.87; **Supplementary Fig. 1** ). The study flow and matched sample sizes (27 vs. 81) are shown in Fig. 1 .

All statistical analyses were performed using EZR (Saitama Medical Center, Jichi Medical University, Saitama, Japan), which serves as a graphical user interface for R (The R Foundation for Statistical Computing, Vienna, Austria). EZR is a modified version of R Commander that incorporates additional statistical functions commonly used in biostatistics 17 . Continuous variables are presented as the median with interquartile range (IQR) and were compared using the Mann–Whitney U test. Categorical variables are expressed as counts and percentages, and intergroup comparisons were performed using Fisher’s exact test. *P* < 0.05 was considered statistically significant.

## Results

### Baseline clinical characteristics of the patients

Table 1 summarizes baseline clinical characteristics of the GC/IM group (n = 32) and the standard group (n = 379). Significant differences were observed in sex, age, and ASA-PS between the groups. Compared with the standard group, the GC/IM group included fewer male patients (46.9% vs. 76.2%, *P* **<** 0.001), had a higher median age 78 years (IQR 75–81) vs. 73 years (IQR 67–79) ( *P* = 0.010), and had a markedly greater proportion of patients with an ACCI ≥ 4 (84.4% vs. 48.0%, *P* < 0.001) and ASA-PS ≥ 2 (100% vs. 23.7%, *P* < 0.001). Other variables, including BMI, antithrombotic agent use, lesion size, lesion location, lesion multiplicity, procedure time, submucosal fibrosis, muscle layer injury, and use of PGA with fibrin glue, did not differ significantly between the two groups.

**Table:** **Table 1** Clinical characteristics of patients in the GC/IM and standard groups.

**Variables**	**GC/IM group (n = 32)**	**Standard group (n = 379)**	***P* value **
Patient characteristics			
Age	78 (75–81)	73 (67–79)	0.010
Sex (male)	15 (46.9%)	289 (76.2%)	0.001
BMI	21.6 (19.0–24.4)	22.7 (20.6–24.6)	0.134
ACCI (≥ 4)	27 (84.4%)	182 (48.0%)	< 0.001
ASA-PS (≥ 2)	32 (100%)	90 (23.7%)	< 0.001
Antithrombotic agents	10 (31.0%)	92 (24.2%)	0.406
NSAID daily use	3 (9.4%)	16 (4.2%)	0.177
Lesion characteristics			
Lesion size	16 (10–26)	15 (10–25)	0.697
Location (Upper)	18 (56.3%)	205 (54.1%)	0.236
Multiplicity of lesions	5 (15.6%)	53 (14.0%)	0.792
Procedure details			
Submucosal fibrosis	6 (18.8%)	51 (13.5%)	0.423
Injury to the muscle layer	3 (9.4%)	40 (10.6%)	1.000
Operator skill (expert)	3 (9.4%)	93 (24.5%)	0.052
Procedure time	62 (42–120)	85 (55–130)	0.161
PGA with fibrin glue	6 (18.7%)	38 (10.0%)	0.130
ACCI, age-adjusted Charlson Comorbidity Index; ASA-PS, American Society of Anesthesiologist Physical Status; GC, glucocorticoid; IM, immunomodulator; PGA, polyglycolic acid sheet. Fisher`s exact test was used for categorical variables and the Mann-Whitney U test was applied for non-categorical variables.			

Within the GC/IM group, 30 patients (93.7%) received GC, seven patients (21.8%) received GCs and IMs, and two patients (6.3%) received IMs alone. Median daily GC dose was 5 mg (IQR 4–5), with a maximum dose of 10 mg/day. Among the IMs, methotrexate was most frequently prescribed (six patients, 60.0%), followed by tacrolimus (two patients, 20.0%), a combination of methotrexate and tacrolimus (one patient, 10.0%), and azathioprine (one patient, 10%).

In the GC/IM group, rheumatoid arthritis was the most prevalent comorbidity (43.8%), followed by polymyalgia rheumatica (12.5%) and systemic lupus erythematosus (9.4%). Other autoimmune and inflammatory conditions, including membranous nephropathy, microscopic polyangiitis, autoimmune pancreatitis, mixed connective tissue disease, immunoglobulin A nephropathy, giant cell arteritis, psoriasis, sarcoidosis, and scleroderma, collectively affected 34.4% of patients ( Table 2 ).

**Table:** **Table 2** Comorbidities in patients in the GC/IM group.

**Comorbidity**	**Number of cases**	**Percentage (%)**
Rheumatoid arthritis	12	43.8
Polymyalgia rheumatica	4	12.5
Systemic lupus erythematosus	3	9.4
Others	13	34.4
Total	32	100
GC, glucocorticoid; IM, immunomodulator.		

### Propensity score matching

Table 3 shows clinical characteristics of the GC/IM group and the standard group after propensity-score matching (PSM). Following matching, the GC/IM group (n = 27) and the standard group (n = 81) showed good balance across most key variables. For instance, the ASD for sex decreased from 0.630 before matching to 0.075 after matching, and the ASD for the ACCI declined from 0.830 to 0.100. Although the ASD for age and ASA-PS remained above the ideal threshold of 0.100, other variables, such as use of antithrombotic agents (ASD: 0.060) and lesion size (ASD: 0.080), demonstrated good balance. These findings indicate that PSM successfully minimized potential confounding factors, resulting in a more balanced comparison between the groups.

**Table:** **Table 3** Clinical characteristics of patients in the GC/IM and standard groups after propensity score matching.

	**Propensity-score matched cases (n = 108)**			
**Variable**	**GC/IM (n = 27)**	**Standard (n = 81)**	***P* value **	**ASD**
Patient characteristics				
Age (≥ 65)	23 (85.2)	80 (98.8)	0.013	0.520
Sex (male)	15 (55.6)	42 (59.3)	0.823	**0.075**
BMI (≥ 21)	18 (66.7)	57 (70.4)	0.810	0.080
ACCI (≥ 4)	22 (81.5)	69 (85.2)	0.761	**0.100**
ASA-PS (≥ 2)	27 (100)	69 (85.2)	0.035	0.590
Antithrombotic agents	8 (30.8)	16 (20.2)	0.290	**0.060**
NSAID daily use	3(11.1)	8(9.9)	1.000	0.040
Lesion characteristics				
Lesion size (≥ 21)	8 (29.6)	27 (33.3)	0.815	**0.080**
Location (Upper)	3 (11.1)	6 (7.4)	0.688	**0.128**
Multiplicity of lesion	4 (15.4)	10 (12.8)	0.750	**0.074**
Procedure details				
Submucosal fibrosis	5 (18.5)	14 (17.3)	1.000	0.032
Injury to the muscle layer	3 (11.1)	9 (11.1)	1.000	< 0.001
Operator skill (Expert)	2 (7.4)	18 (22.2)	0.150	0.426
Procedure time (≥ 61)	15 (55.6)	55 (67.9)	0.255	**0.256**
PGA with fibrin glue	3 (11.1)	6 (7.4)	0.688	**0.128**
ACCI, age-adjusted Charlson Comorbidity Inex; ASA-PS, American Society of Anesthesiologists physical status; ASD, absolute standardized difference; GC, glucocorticoid; IM, immunomodulator; NSAID, nonsteroidal anti-inflammatory drug; PGA, polyglycolic acid.				

### Primary and secondary outcomes after propensity score matching

Table 4 presents primary and secondary outcomes for the GC/IM group and the standard group after PSM. The overall post-ESD complication rate was significantly higher in the GC/IM group than in the standard group (44.4% vs. 21.0%, *P* = 0.024; relative risk [RR] 2.11; 95% CI 1.16–3.84). Specifically, post-ESD pain (40.7% vs. 18.5%, *P* = 0.035; RR 2.2; 95% CI 1.15–4.19) and post-ESD fever (11.1% vs. 1.2%, *P* = 0.047; RR 9.0; 95% CI 0.98–82.9) occurred more frequently in the GC/IM group. By contrast, there were no significant differences between the two groups in rates of delayed bleeding (3.7% vs. 2.5%, *P* = 1.000), delayed perforation (0.0% vs. 0.0%, *P* = 1.000), or intraoperative perforation (7.4% vs. 3.7%, *P* = 0.162). Similarly, median length of hospital stay did not differ significantly between the two groups (9 days [IQR 7–10] vs. 8 days [IQR 7–9]; *P* = 0.064). Regarding laboratory data, the standard group showed a significantly greater change in logarithmic WBC count (0.09 vs. 0.17, *P* = 0.022), although changes in logarithmic C-reactive protein concentration did not differ significantly between the two groups (0.54 vs. 0.65, *P* = 0.668).

**Table:** **Table 4** Post-ESD outcomes after propensity-score matching (GC/IM vs standard): complications, hospital stay, labs, and management.

**Variable**	**GC/IM (n = 27)**	**Standard (n = 81)**	**RR (95%CI)**	***P* value **
Complications				
Overall complications	12 (44.4)	17 (21.0)	2.1 (1.2–3.9)	**0.024**
Post ESD pain	11 (40.7)	15 (18.5)	2.2 (1.2–4.2)	**0.035**
Post ESD fever	3 (11.1)	1 (1.2)	9 (0.98–83)	**0.047**
Delayed bleeding	1 (3.7)	2 (2.5)	1.5 (0.14–16)	1.000
Delayed perforation	0 (0)	0 (0)	NA	1.000
Intraoperative perforation	2 (7.4)	3 (3.7)	2 (0.35–11)	0.162
Resource use				
Hospital stay	9 (7–11)	8 (7–9)	NA	0.064
Second look endoscopy	2 (7.4)	3 (3.7)	2 (0.35–11)	0.597
Laboratory changes				
△log-CRP	0.54 (0.15–1.17)	0.65 (0.35–0.95)	NA	0.668
△log-WBC	0.09 (0.02–0.2)	0.17 (0.08–0.25)	NA	**0.022**
Symptom management				
Analgesic use	7 (25.9)	3 (3.7)	4.5 (1.8–11)	**0.002**
Antipyretic use	2 (7.4)	2 (2.5)	3 (0.44–20)	0.270
Antibiotic use	2 (7.4)	3 (3.7)	2 (0.35–11)	0.597
CRP, C-reactive protein; ESD, endoscopic submucosal dissection; GC, glucocorticoid; IM, immunomodulator; WBC, white blood cell.				

**Fig. 2:**
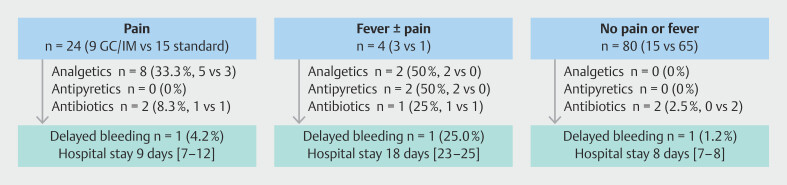
Post-ESD symptom subgroups and subsequent management/outcomes in the propensity-matched cohort. Patients were stratified into three symptom categories after gastric ESD: 1) abdominal pain only; 2) fever ± pain; and 3) no pain or fever. For each subgroup, the chart displays counts of patients requiring antibiotics, antipyretics, or analgesics (shown as total % and GC/IM vs standard counts) and downstream outcomes—delayed bleeding and median [IQR] length of stay. Arrows illustrate the descriptive flow from symptoms → management → outcomes and do not imply causality. GC/IM, glucocorticoid or immunomodulator; IQR, interquartile range.

On-demand analgesics for pain were given to 25.9 % of GC/IM patients vs 3.7% of controls ( *P* = 0.002), antipyretics for fever to 7.4% vs 2.5% ( *P* = 0.270), and antibiotics to 7.4% vs 3.7% ( *P* = 0.597) ( Table 4 ). To examine how early symptoms influenced subsequent treatment requirements and clinical course, we grouped patients according to initial symptom burden. In the pain-only subgroup (n = 24), analgesics were administered in eight of 24 (33 %) and median hospital stay was 9 days (IQR 7–12); in the fever ± pain subgroup (n = 4), antipyretics and analgesics were each used in two of four (50%) and median hospital stay was 18 days (IQR 13–25); no-symptom patients (n = 80) required neither analgesics nor antipyretics and stayed a median of 8 days (IQR 7–8) ( Fig. 2 ).

Finally, an exploratory look within the GC/IM cohort showed no clear pattern by agent type (overall complications: GC only 50%, GC + IM 40 %, IM only 0%; *P* = 0.389) or by GC dose (≤ 5 mg 57% vs > 5 mg 0%; *P* = 0.096), and no individual outcome reached significance ( **Supplementary Table 1** , **Supplementary Table 2** ).

## Discussion

This study evaluated the impact of GC and IM on risk of post-ESD complications. The findings showed that patients receiving GC/IM had a significantly higher overall post-ESD complication rate than the standard group. However, the increase in post-ESD complications in the GC/IM group was attributed to minor complications, such as abdominal pain and fever, but not to severe complications, such as delayed bleeding and perforation. Therefore, ESD appeared to be a clinically feasible option for immunocompromised individuals, provided that early warning signs such as abdominal pain were closely monitored and appropriately managed.

We chose a composite primary endpoint because even “minor” symptoms—pain or fever—immediately prompt additional monitoring, imaging, or analgesic use. Combining these frequent actions with rarer major complications provides the most sensitive gauge of total post-ESD workload and matches our a priori hypothesis that overall events might rise while catastrophic events remain uncommon.

Abdominal pain is one of the most common complications associated with gastric ESD. The inflammatory response after mucosal injury is considered a primary cause of such pain 18 , involving activation of intramuscular sensory nerves by mechanical stimulation from mucosal defects, thermal injury, and peritoneal irritation during the ESD procedure. The higher frequency of abdominal pain observed in the GC/IM group may be explained by the fact that chronic GC/IM therapy can dampen the normal inflammatory cascade and impair tissue repair, potentially leading to atypical or prolonged postoperative inflammation 19 . This may manifest as more pronounced or persistent pain, because deeper tissue injury is not fully contained by an efficient immune response.

Fever is another common complication associated with gastric ESD. The inflammatory response due to mucosal injury is also a primary cause of fever 20 , typically involving a localized inflammatory response to thermal injury rather than bacterial infection or bacteremia under normal conditions 20 21 , and it usually resolves with conservative management 22 . Extensive submucosal dissection accompanied by thermal injury can trigger release of inflammatory cytokines through activation of macrophages, endothelial cells, and the reticuloendothelial system 23 . In the GC/IM group, subclinical bacterial infection and/or bacterial translocation may be more likely due to immunosuppression, potentially contributing to increased frequency of post-gastric ESD fever observed in this study.

Although abdominal pain and fever associated with gastric ESD were more common in the GC/IM group, major complications such as delayed bleeding and perforation were not increased. Routine use of P-CABs and prophylactic use of PGA sheets may have contributed to reduced risk of these serious complications, particularly in the GC/IM group. P-CABs have been reported to help prevent delayed bleeding 24 , and prophylactic use of PGA sheets is known to support the healing process 25 . In addition, we made every effort to perform gastric ESD using a meticulous technique to minimize thermal injury to the muscular layer. As a result, incidence of abdominal pain in both the standard group and the GC/IM group was lower than the previously reported rates of 53.8% to 98% 18 26 . Similarly, incidence of fever in both groups was also lower than the previously reported rates of 23.1% to 24.8% 22 27 . A large-scale, multicenter, prospective study is needed to validate our observation that incidence of major complications, including delayed bleeding and/or perforation, is not increased in patients receiving GC/IM treatment.

In addition, several endoscopic closure techniques such as clipping devices, endoscopic suturing, and over-the-scope clips have been developed recently 28 . Although our study employed prophylactic PGA sheets and fibrin glue, robust evidence demonstrating their effectiveness in preventing perforation remains limited. Future studies should investigate the efficacy of these various closure techniques.

By contrast, incidence of intraprocedure perforation was numerically higher in the GC/IM group, although the difference was not statistically significant ( *P* = 0.162) and was based on only five events in total; therefore, a modest drug-related effect cannot be excluded, and these findings should be interpreted with caution.

Although our data confirm a higher rate of minor events in GC/IM patients, these events were effectively managed with on-demand medication and close observation, supporting the notion of clinical—rather than universal—feasibility. Although the overall median length of stay differed by only 1 day (9 days [7–12] in GC/IM vs 8 days [7–9] in controls), the small fever ± pain subgroup stayed much longer (18 days [13–25]). Minor symptoms, therefore, added a modest workload in most cases, yet occasionally required substantial extra observation. Consistent with this pattern, on-demand medication use was three- to seven-fold higher in GC/IM patients (analgesics 25.9% vs 3.7%; antipyretics 7.4% vs 2.5%), reflecting the additional bedside care triggered by pain and fever. Importantly, this burden remained manageable: a proactive, symptom-directed protocol contained resource use to extra medication and monitoring without increasing serious AEs or extending hospitalization beyond what symptoms dictated.

Beyond overall GC/IM exposure, we explored whether specific agents or doses modified risk; no clear agent‑ or dose‑dependent signal emerged. Complication rates were similar in GC‑only users (50%) and in GC+IM users (40%) and were absent in the two IM‑only cases ( *P* = 0.389). Dose stratification likewise showed 57% complications with≤5mg GC vs none with > 5 mg ( *P* = 0.096). The limited sample sizes in certain subgroups constrain the precision of these estimates.

These clinical symptoms observed in the GC/IM group were not mirrored in the next-day laboratory findings; WBC count tended to be lower in the GC/IM group than in the standard group, likely owing to pharmacologic immunosuppression masking or delaying the typical inflammatory response. Tailored care is essential for managing minor events and for preventing escalation into more serious adverse outcomes.

This study has several limitations. As a single-center, retrospective cohort study, generalizability of the findings is limited, and the small sample size, particularly in the GC/IM group, reduced the statistical power. In addition, patients at higher risk of perforation or those with lesions in anatomically challenging locations may have been preferentially directed toward surgery rather than ESD, introducing potential selection bias. We included only patients receiving GC doses ≤ 10 mg/day, without accounting for treatment duration or cumulative dosage. As a result, the safety of ESD in patients on higher doses or prolonged GC therapy remains unclear. Future research with larger cohorts and varying GC doses is needed to provide a more comprehensive assessment. Finally, matching for the ACCI was imperfect (ASD: 0.100), reflecting the difficulty of identifying comparable controls for GC/IM patients with complex comorbidities. Despite these limitations, PSM offered a reasonable approach to evaluate the impact of glucocorticoids and immunomodulators on gastric ESD outcomes.

## Conclusions

In patients receiving glucocorticoids or immunomodulators, gastric ESD was associated with increased incidence of minor post-procedural complications—chiefly abdominal pain and transient fever—leading to greater use of symptom-directed medication and slightly longer observation. Nonetheless, with careful technique, vigilant postoperative monitoring, and prompt symptom management, these minor events were readily controlled and major complications remained uncommon. Thus, gastric ESD appeared clinically feasible in this immunosuppressed population when undertaken with proactive management strategies.

## Data Availability Statement

All data used in this study are securely stored at Kyushu Medical Center. Access to these data may be granted upon reasonable request to the corresponding author or the institutional review board, in accordance with applicable regulations and institutional guidelines.

## Notes

Correction **Clinical feasibility of gastric endoscopic submucosal dissection in patients on glucocorticoids or immunomodulators: Propensity-score-matched study** Hiroki Fukuya, Eikichi Ihara, Yoichiro Iboshi et al. Endoscopy International Open 2025; 13: a27331229. DOI: 10.1055/a-2733-1229 In the above-mentioned article the affiliation of authors Akihisa Ohno and Mitsuru Esaki as well as figure 2 were corrected. The affiliation of authors Akihisa Ohno and Mitsuru Esaki was corrected to: Department of Medicine and Bioregulatory Science, Graduate School of Medical Sciences, Kyushu University, Fukuoka, Japan. Fig. 2 was corrected. This was corrected in the online version on 9 March 2026.

## Supplementary Material

Supplementary Material Zusatzmaterial
