# Correction: Clinical feasibility of gastric endoscopic submucosal dissection in patients on glucocorticoids or immunomodulators: Propensity-score-matched study

**DOI:** 10.1055/a-2823-6852

**Published:** 2026-03-11

**Authors:** Hiroki Fukuya, Eikichi Ihara, Yoichiro Iboshi, Yorinobu Sumida, Daisuke Yoshimura, Shohei Hamada, Taisuke Sasaki, Akito Ohkubo, Shuichi Itonaga, Hitoshi Homma, Ryota Okitsu, Akihisa Ohno, Mitsuru Esaki, Naohiko Harada

**Affiliations:** 1 37085 Department of Gastroenterology, National Hospital Organisation Kyushu Medical Center, Fukuoka, Japan; 2 Institute of Research Center, National Hospital Organization Kyushu Medical Center, Fukuoka, Japan; 3 12923 Department of Medicine and Bioregulatory Science, Graduate School of Medical Sciences, Kyushu University, Fukuoka, Japan; 4 Department of Gastroenterology, Kitakyushu Municipal Medical Center, Fukuoka, Japan



In the above-mentioned article the affiliation of authors Akihisa Ohno and Mitsuru Esaki as well as figure 2 were corrected.

The affiliation of authors Akihisa Ohno and Mitsuru Esaki was corrected to: Department of Medicine and Bioregulatory Science, Graduate School of Medical Sciences, Kyushu University, Fukuoka, Japan.

 This was corrected in the online version on 9 March 2026.

