# Supplementary material for: Clinical feasibility of gastric endoscopic submucosal dissection in patients on glucocorticoids or immunomodulators: Propensity-score-matched study
Source: Endosc Int Open. 2025 Nov 11;13:a27331229. doi: 10.1055/a-2733-1229 (PMC12690568; doi:10.1055/a-2733-1229)

Supplementary Table 1 Subgroup analysis of overall post-ESD complications and hospital stay in the GC/IM Cohort, classified by agent type.

Variable	GC-only (n = 20)	Combination (n = 5)	IM only (n = 2)	*P* value
Overall complications	10 (50)	2 (40)	0 (0)	0.389
Post ESD pain	9 (45)	2 (40)	0 (0)	0.466
Post ESD fever	3 (15)	0 (0)	0 (0)	0.554
Delayed bleeding	1 (5)	0 (0)	0 (0)	0.834
Intraoperative perforation	1 (5)	0 (0)	1 (50)	0.053
Hospital stay	9 [7-12]	9 [7-9]	9 [8-9]	0.856

Fisher's exact test was used for categorical variables and Kruskal–Wallis test was applied for non-categorical variables. Categorical variables were expressed as counts and frequencies, non-categorical variables were expressed as median, interquartile range (IQR). ESD, endoscopic submucosal dissection; GC, glucocorticoid; IM, immunomodulator.

Supplementary Table 2 Subgroup analysis of post-ESD complications and hospital stay in the GC/IM Cohort, classified by GC dose.

Variable	GC low (n = 21)	GC high (n = 4)	*P* value
Overall complications	12 (57.1)	0 (0)	0.096
Post ESD pain	11 (52.4)	0 (0)	0.110
Post ESD fever	3 (14.3)	0 (0)	1.000
Delayed bleeding	1 (4.8)	0 (0)	1.000
Intraoperative perforation	1 (4.8)	0 (0)	1.000
Hospital stay	8 [7-10]	11 [9-12]	0.420

ESD, endoscopic submucosal dissection; GC, glucocorticoid; IM, immunomodulator.

Supplementary Figure 1 Supplemental Figure. An area under the receiver operating characteristic curve (AUROC) 0.79 (95% CI, 0.70–0.87), confirming moderate validity in predicting GC/IM use.

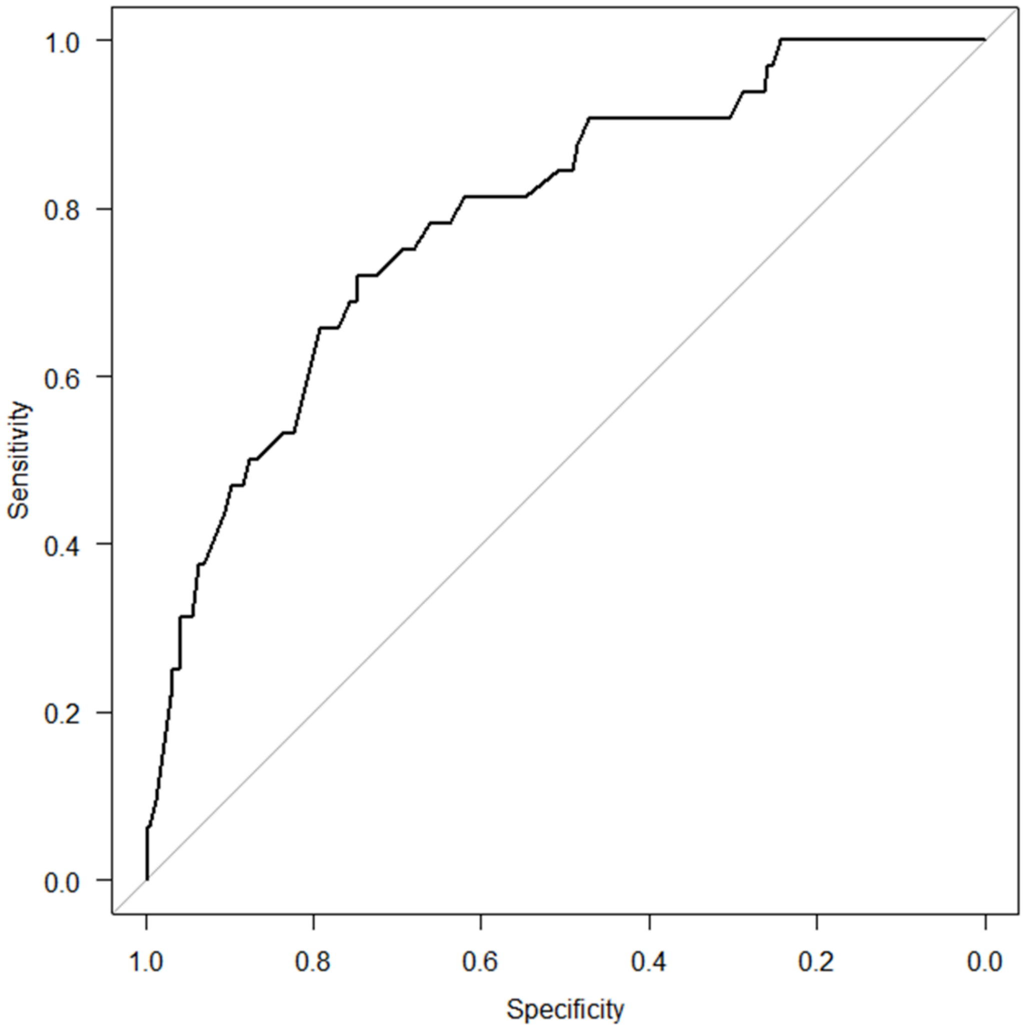

Supplement: Supplementary file 1 — Supplementary Material [file 10-1055-a-2733-1229_27355842.pdf]
